# Atmospheric blockages as trigger of environmental contingencies in Mexico City^☆^

**DOI:** 10.1016/j.heliyon.2019.e02099

**Published:** 2019-07-24

**Authors:** Rafael Silva-Quiroz, Ana Leonor Rivera, Paulina Ordoñez, Carlos Gay-Garcia, Alejandro Frank

**Affiliations:** a Posgrado en Ciencias de la Tierra, Universidad Nacional Autónoma de México, Circuito Exterior, Ciudad Universitaria, Mexico City, 04510, Mexico; b Centro de Ciencias de la Complejidad, Universidad Nacional Autónoma de México, Circuito Mario de la Cueva 20, Insurgentes Cuicuilco, Mexico City, 04510, Mexico; c Instituto de Ciencias Nucleares, Universidad Nacional Autónoma de México, Circuito Exterior, Ciudad Universitaria, Mexico City, 04510, Mexico; d Centro de Ciencias de la Atmósfera, Universidad Nacional Autónoma de México, Circuito de la Investigación Cientifica, Ciudad Universitaria, Mexico City, 04510, Mexico

**Keywords:** Environmental sciences, Atmospheric science, Zone, Atmospheric blocking, Atmospheric composition, Environmental contingencies, Meteorology, Statistics, Atmospheric chemistry, Air quality, Environmental analysis, Environmental pollution

## Abstract

Atmospheric pollution in cities is due to several human factors, for instance the number of cars in circulation, fuel efficiency and industrial waste, as well as orographic and meteorological conditions that determine air circulation. Ozone contingencies cause health disorders on the population, making it important to understand the factors that trigger such contingencies. Here, we analyze meteorological (wind, temperature, relative humidity) and atmospheric composition (ozone, and NOx) data of five atmospheric monitoring stations on Mexico City, from March 2004 to May 2018, comparing normal days with the extreme days in the 90th percentile of ozone. Moreover, we present the synoptic patterns of the seasonal differences of geopotential height at 500 hPa between extreme and control days. We found that, in the dry-hot season (from March to May) an atmospheric blockage with meteorological conditions of almost no wind, low relative humidity, and small temperature fluctuations occurs. Because the air in the city permanently contains large amounts of ozone precursors like NOx, this meteorological scenario raises ozone levels to those of an environmental contingency. Thus, during the dry-hot season on Mexico City, ozone contingencies are triggered by atmospheric blocking. This scenario will be present in cities surrounded by mountains with high levels of Ozone precursors.

## 1 Introduction

Air pollution is a serious health problem, one that according to the World Health Organization (WHO) causes 3.7 million deaths worldwide annually (WHO, 2018), many of which are associated with respiratory diseases (Grigg, 2018), (Falcon-Rodriguez et al., 2017), but also with cardiac problems due to an autonomic nervous systems dysfunction (Buteau and Goldberg, 2016). Between the principal contaminants we found the tropospheric ozone, recognized as the second pollutant most detrimental to human health (Ebi and McGregor, 2008), and vegetation (Zhou et al., 2018). Roughly 5–20% of air pollution-related deaths are estimated to be linked to ozone (Anenberg et al., 2009), (Brauer et al., 2012). Ozone is also detrimental to crops and ecosystems health, impeding the uptake of carbon into the biosphere (Monks et al., 2015). It is also a greenhouse gas, so its regulation has an important role in climate change mitigation (IPCC, 2013). Due to these, it has been identified as one of the principal trigger factors of pollution contingencies.

Ozone is not emitted directly into the atmosphere, it is produced by photo-chemical mechanisms from a wide variety of natural and anthropogenic precursors such as non-methane volatile organic compounds (VOC), carbon oxides (CO_*x*_), and nitrogen oxides (NO_*x*_), mainly emitted at the surface by combustion processes (Cohen et al., 2018). The tropospheric ozone concentration at any given location depends on the proximity to large sources of ozone precursors, the prevailing meteorological conditions, the stratosphere-troposphere exchange, and the long-range atmospheric transport (Lefohn et al., 2018). Overall, the emissions from vehicles dominate as a major source of ozone precursors in many cities (Monks et al., 2015), in particular in Mexico city (Paniagua et al., 2017).

An air quality monitoring network of Mexico City was established in 1986, and has helped to document the evolution of some pollutants following the implementation of various emission-reduction programs (INE, 1998). The governmental measures have consisted in the creation of a program to limit the number of vehicles circulating -Programa Hoy No Circula- (PHNC). However, this measure has been widely criticized for not significantly improving air quality (Davis, 2008). Similar measures have been applied in the world. Beijing, for example, has attempted to reduce emissions and improve air quality before major events such as the Olympic Games (Wang et al., 2010). The principal problem with this type of measures is that it affects the mobility of the population and therefore the local economy, and it is not clear if they are efficient at reducing pollution levels. On March, 2006, the Megacity Initiative: Local and Global Research Observations (MILAGRO) measurement field campaign in Mexico City identified that any reduction in VOC emissions led to a decrease in the maximum ozone concentrations in the urban area of the city (Song et al., 2010). Other authors (Stephens et al., 2008) reported that only large reductions in NO_*x*_ emissions could effectively reduce ozone in the urban area of Mexico City. This behavior has been identified as characteristic of extra-tropical megacities in the Northern Hemisphere (Monks et al., 2015). In Mexico City this effect has become more pronounced in recent years because there is a significant reduction of CO and VOC emissions, but relatively steady NO_*x*_ emissions (Song et al., 2010).

Mexico's government declared an environmental contingency when a certain ozone concentration threshold is exceeded: in the 1990–2006 period the threshold was at 256 ppb, 221 ppb in the 2006–2012 period, 194 ppb in the 2012–2016 and 155 ppb since 2016 until the time this paper was written (Aire, 2018). These thresholds were based on epidemiological measurements determined by the incidences in respiratory diseases (Diario Oficial de la Federacion, 2014). However, doing this, the results can be skewed. To avoid this, in this paper, from now on, environmental contingencies will be referred to events in which the ozone exceeds the 90th percentile levels (determined from data between 2004 and 2018, where the Ozone levels remained relatively controlled).

It is of great interest to determine when there will be an ozone environmental contingency in Mexico City. Ozone precursors in the city have concentrations that never become null, thus, our hypothesis is that meteorological factors could trigger ozone contingencies. Some studies have established the connection of tropospheric ozone with circulation patterns (e.g. Demuzere et al., 2009; Carvalho et al., 2010; Stock et al., 2014). In Mexico City, past studies noted a primary relationship between ozone concentration in Mexico City and ultraviolet (UV) radiation, where days with more UV radiation were associated with elevated surface ozone concentrations (Raga and Le Moyne, 1996). Other authors (Barrett and Raga, 2016) studied the synoptic-scale patterns associated with days of low and high ozone concentrations in winter and summer during the period (1986–2014). However, the highest ozone concentrations occurred in spring when the Mexico City area is characterized by intense solar radiation and high temperature which influence the ozone concentrations (ITF, 2017), which are nearly 30% higher than in the rest of the year(Barrett and Raga, 2016). The meteorological triggers and the synoptic patterns associated to these ozone contingencies are not yet fully understood.

The main objective of this study is to identify the meteorological factors and synoptic patterns leading to extreme ozone events in the metropolitan area of Mexico City during the sunny and hot spring. This article is organized as follows. Section 2 describes the data used as well as the methodology applied. In section 3 the main results are presented and discussed. Finally, conclusions are given in Section 4.

## 2 Data and methods

### 2.1 Local data time series

In order to determine factors that unleash environmental contingencies in the metropolitan area of Mexico City, meteorological data was analyzed: temperature (T), wind speed (WSP) and relative humidity (RH), as well as air pollutant data: Nitrogen oxides (NO_*x*_), and ozone (O_3_). The data used was obtained from the environmental government network of the city -Red Automática de Monitoreo Atmosférico, RAMA, the description of the acquisition methodology is detailed on http://www.aire.cdmx.gob.mx/default.php.

We consider only five RAMA stations due to the quality of the data (less than 10% in missing data) and because their records go back to 1986:

– San Agustin (SAG) at 2241 m height, latitude −99.030324, longitude 19.532968,
– Tlalnepantla (TLA) at 2311 m height, latitude −99.204597, longitude 19.529077,
– Merced (MER) at 2245 m height, latitude −99.119594, longitude 19.424610,
– UAM Iztapalapa (UIZ) at 2221 m height, latitude −99.073880, longitude 19.360794, and
– Pedregal (PED) at 2326 m height, latitude −99.204136, longitude 19.325146.

**Figure 1:**
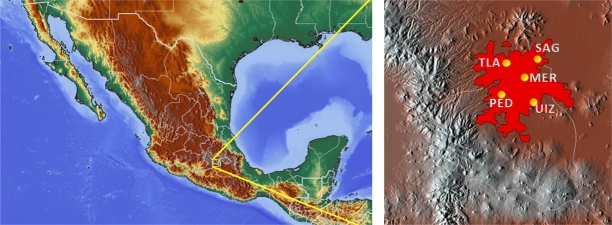
Locations of RAMA measurement surface environmental monitoring stations used in this work in a orographic map. Metropolitan area is shown in red in the right image.

These stations are localized in the orographic map of Fig. 1. Data on these stations is recorded hourly.

In this work, the 90th local percentile at each of the five urban sites was defined as the threshold for the ozone contingency events: for MER 133 ppb of ozone concentration; SAG 113; UIZ 135; TLA 124; and PED 148. Time series for the daily maximum ozone values of each station were transformed to a binary format, if the value exceeded the 90th percentile, the number one was assigned, otherwise the number was set to zero. If in a single day three of the five stations added a value greater than or equal to 3, the day was considered as an ozone contingency event. Once the events were identified, the 24 hours prior to that maximum were extracted from the data set in all the variables. In this way, by combining all the cases, a composite can be created. In the results section, the patterns observed in each variable will be discussed.

We also used historic Upper-air soundings at the Mexico City International Airport (MEX; elevation 2230 m) to analyze the mid-troposphere wind velocity over the city in the period 2004 to 2018. These soundings were obtained from the University of Wyoming (http://weather.uwyo.edu/ upperair/sounding.html). We confine our analysis to just afternoon soundings (0000 UTC; 1700 local time). Climate Hazards Group InfraRed Precipitation (CHIRPS) database (Funk et al., 2015) was also used for the grid point in which Mexico City is located. This database integrates 0.05° resolution satellite imagery with in-situ station data and it was shown by Perdigón-Morales et al. (2018) to properly reproduce the precipitation over Mexico.

The data used to compute these composites was obtained from the ERA-Interim reanalysis for the period of 2004–2017 (Dee et al., 2011). Daily values were obtained by averaging the 6-hourly data of geopotential heights at 500 hPa and potential temperature at the dynamical tropopause at $1^{o}\times 1^{o}$ horizontal resolution.

## 3 Results and discussion

### 3.1 Ozone concentration in Mexico City

**Figure 2:**
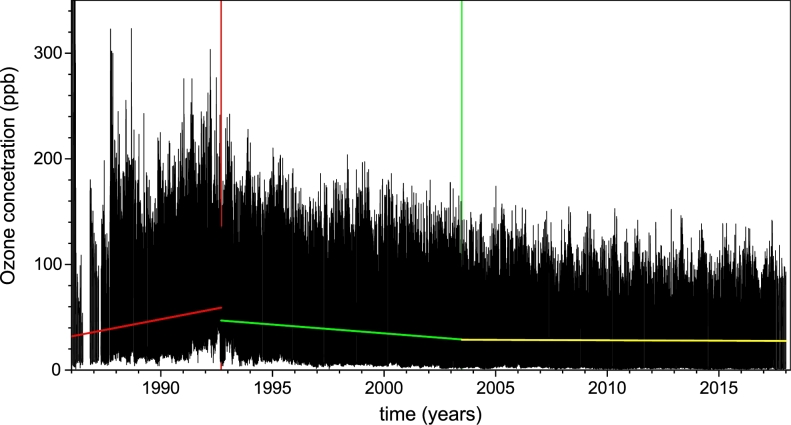
Atmospheric ozone concentration on Mexico city from January 1986 to June 2018. Minimum squares best linear fit found three tendencies: an increasing tendency with a slope of (4.1 ± 0.1) ppb/h from January 17, 1986 to September 14, 1992 (red line), a decreasing one with a slope of (−1.67 ± 0.04) ppb/h from September 14, 1992 to June 25, 2003 (green line), and since then, an almost constant behavior with a slope of (−0.14 ± 0.02) ppb/h (yellow line).

Average of the tropospheric ozone concentration in the five Mexico City RAMA stations taken hourly is plotted in Fig. 2. In the plot can be seen three distinctive regions of increasing, decreasing and of constant tendency. By mean squares adjustment of the data, we found for each region the best linear adjustment to the data (maximum Pearson's r, minimum $R^{2}$):

– from January 17, 1986 to September 14, 1992, we found an increasing tendency with a slope of (4.1±0.1) ppb/h, leading to ozone levels that surpassed those suggested by the World Health Organization (WHO, 2018),
– from September 14, 1992 to June 25, 2003, there was a decreasing tendency with a slope of (−1.67±0.04) ppb/h. In this region ozone concentration was still above healthy levels, for most of the year,
– from June 25, 2003 to June 1, 2018, an almost constant average tendency was found, with a slope of (−0.14±0.02) ppb/h.

In this paper we limit our analysis to the region of average constant trend.

**Figure 3:**
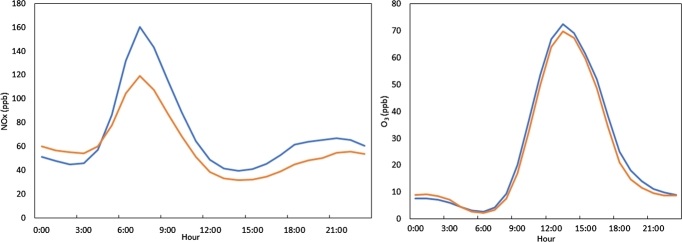
Average NO_*x*_ (left panel) and ozone (right panel) concentrations as function of the hour of the day on weekends (orange line) and from Monday to Friday (blue line). Data from UNAM meteorological station average values from January 2004 until June 2018.

Before the middle of the 90's, Particulate Matter (PM) was the most common pollutant due to the industrial activity within the city (Soto-Coloballes, 2017). In addition, the levels of lead in the environment became critical, so the chemical additives in gasoline were changed (PROFECO, 2014). These new additives reduced lead but produced greater amounts of NO_*x*_. In this scenario ozone levels during the 1990s surpassed the level suggested by the World Health Organization (WHO, 2018). At the beginning of the 90ś, the highest peak of ozone concentration was reached, and numerous birds died in the city. Fortunately, Mexico City's government implemented drastic measures like taking out many industries from the city, closing the Azcapotzalco Oil Refinery, implementing a change in the composition of gasoline and diesel, a road space rationing program, restrictions on private vehicles circulation, the annual engine verification of vehicles, the change to catalytic conversion in motors, etc. (INE, 1998). With these policies the tendency of ozone concentration started to decrease. However, in the middle of 2003 the city's atmospheric composition reached a steady state with a constant tendency of ozone concentration. Even when NO_*x*_, one of the principal precursors of ozone, diminished in the atmosphere during weekends, the ozone levels has a similar daily profile, independent of the day of the week (see Fig. 3), confirming as shown by others authors (Stephens et al., 2008) the observation that there is always enough precursors to produce ozone. Thus, the tropospheric composition in Mexico City is in a permanent condition that can generate an ozone environmental contingency if a meteorological trigger occurs.

The annual cycle of average surface ozone concentrations (ppb) for the five RAMA stations during the period 2004–2018 are illustrated in the Fig. 1 of supplementary material. In such figure the observations are smoothed using a 30-day running mean. The dry-hot season (which in Mexico corresponds to spring) occurs during the months of March-April-May (MAM). MAM, which is the warmest season in Mexico City, is the time of the year which displays a growing tendency in the levels of ozone. This coincides with what was recently reported for previous years (Retama et al., 2015); (Barrett and Raga, 2016).

Considering all these factors, we focused our study in the data from five RAMA stations (MER, TLA, SAG, UIZ, PED) on the MAM season (March-April-May) from March 2004 to May 2018, defining a contingency day when 3 or more stations have ozone levels above the 90th percentile, as discussed before.

### 3.2 Meteorological factors on contingency events

**Figure 4:**
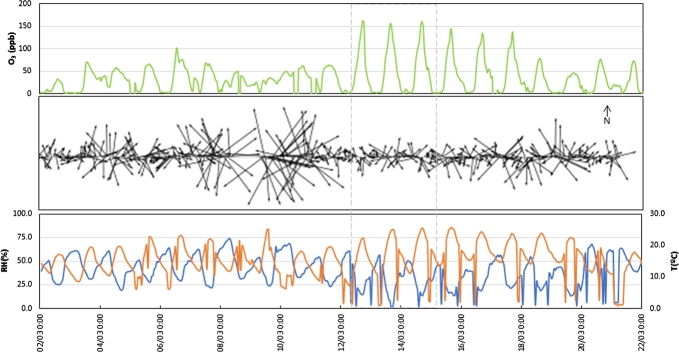
Temporal records before, during and after the Ozone environmental contingency of March 12, 2016 (delimited by the vertical lines). Ozone concentration is in the Upper panel, wind speed and direction are in the middle panel, while lower panel correspond to temperature (orange line) and relative humidity (blue line).

As an example of a typical contingency event on MAM, Fig. 4 shows meteorological variables (temperature, relative humidity, surface wind speed and direction) and ozone concentration before, during and after the ozone contingency of March 12, 2016. It is clearly seen that about one day before the contingency, there are diminished winds, low humidity, and high temperature with less daily fluctuations. These happens in 91% of the contingency events between 2004 to 2018.

**Figure 5:**
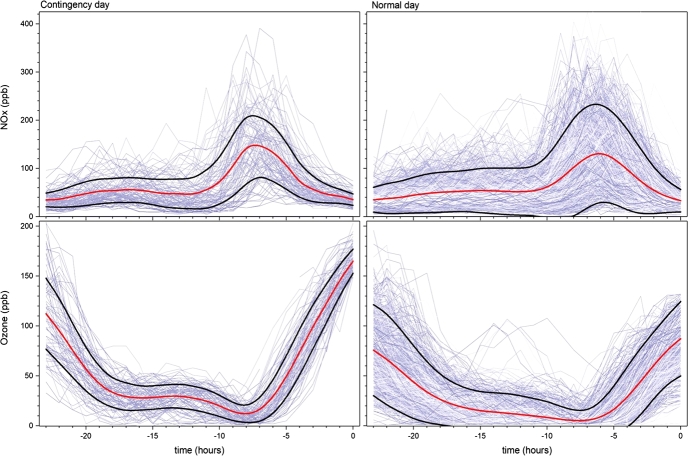
NO_*x*_ (upper panels) and ozone (lower panels) concentrations as function of time for the 24 hours previous to all environmental contingency days (left panels), and normal days (right panels) during MAM from 2004 to 2018. Red line is the mean of all the days on each panel, while black lines correspond to one standard deviation above and under the mean.

**Figure 6:**
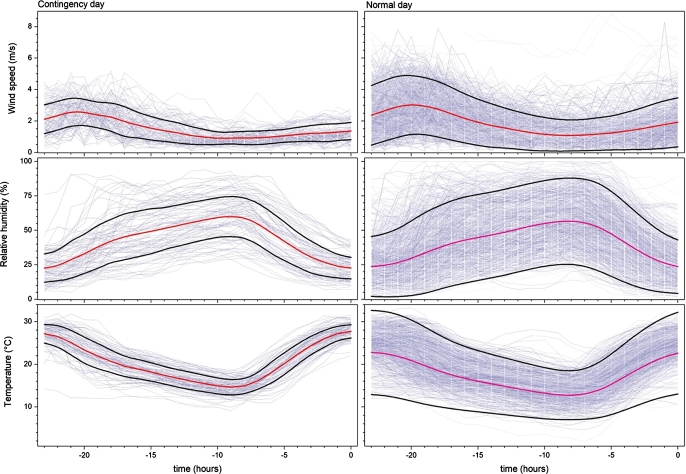
Meteorological factors as function of time for the 24 hours previous to all environmental contingency days (left panels), and normal days (right panels) during MAM from 2004 to 2017. From top to bottom are wind speed, relative humidity and temperature. Red line is the mean of all the days on each panel, while black lines correspond to one standard deviation above and under the mean.

To compare environmental contingency days respect to normal days, composites are plotted with the behavior of each variable shown during the 24 hours prior to an extreme event in the contingency panels at the left-hand of Fig. 5 and Fig. 6, and to normal days selected randomly in the right-hand panels. Each gray line on the plots corresponds to one day, while the red line is the mean of all the days. The black lines correspond to one standard deviation above and under the mean.

Table 1 shows the mean ± standard deviation values for each variable during all the 90th percent extreme events on the MAM period between 2004 and 2018. In the case of environmental contingencies, all the variables show statistically significant less dispersion than on normal days with a significant lower surface wind speed.

**Table 1:** Average values of atmospheric composition and meteorological variables during environmental contingency and normal days.

Variable	Environmental contingencies	Normal days
NO_*x*_ (ppb)	92 ± 40	89 ± 49
Ozone (ppb)	81 ± 12*	45 ± 19

Surface Wind Speed (m/s)	1.76 ± 0.09*	3.1 ± 2
500 hPa Wind Speed (m/s)	5.5 ± 2.3	6.4 ± 4.6
Relative Humidity (%)	48 ± 7*	38 ± 12
Temperature (°C)	25 ± 2*	17 ± 9

* Indicates statistically significant difference.

With respect to the NO_*x*_ precursors (Fig. 5), it is seen that the maximum concentration is around six hours before the ozone maximum. Another maximum corresponds to the emissions of the previous night. Both maxima coincide with the rush hour. For ozone, zero time indicates the time when it is maximum. As expected, the levels decrease to almost zero in the night hours, so a valley-shaped pattern can be seen. Surprisingly, even when NO_*x*_ levels are not statistically significant different between normal and contingency days (Table 1), ozone is higher as expected (by definition contingency days are in the upper 90% of the data).

Regarding the meteorological variables, all are statistically significant different on contingency compared with normal days (see Table 1). An appreciable decrease in surface wind speed (WSP) is observed (see Fig. 6). In the case of relative humidity (RH) there is also a decreasing profile. Otherwise, the temperature (T) is significantly higher. High temperature indicates conditions favorable for photochemical ozone production and the lower wind speed values prevent horizontal dispersion and vertical mixing of air, probably related to stagnation of air masses (Dawson et al. (2008); Garrido-Perez et al. (2018) and references therein).

In fact, a simple Air Stagnation Indices (ASI) that have widely been employed in the literature as a good indicator of episodes of Ozone are based on three meteorological variables: upper-air wind speed, near- surface wind speed and precipitation (Garrido-Perez et al., 2018 and references therein). In our best knowledge an stagnation index has not been established for Mexico City, however, here we observe lower wind speed velocities and daily total precipitation under 1.0 mm (i.e. a dry day) for extreme ozone days.

### 3.3 Synoptic patterns

**Figure 7:**
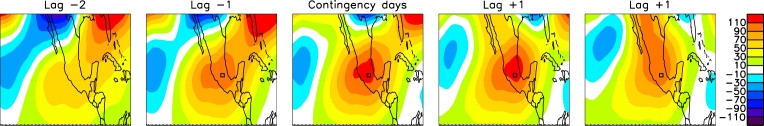
Composites of geopotential height anomalies at 500 hPa (m) for the extreme ozone events (MAM, 2005–2016, middle panel), 2 days before, and 2 days after these events.

Fig. 7 depicts the seasonal differences (MAM) of geopotential height at 500 hPa (Z500) between the ozone percentile 90 (P90) days and the remaining days. The evolution of anomalies is investigated by plotting composites for 2 days before and 2 days after the extreme events. Most extreme ozone events occur under positive anomalies of Z500 which represent slow moving anticyclonic circulations centered over Mexico City which produce subsidence over the region, inhibiting the convection and are favoring the air stagnation.

**Figure 8:**
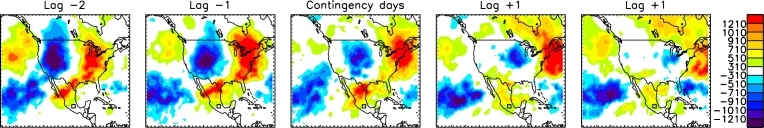
Composites of anomalies of potential temperature on the tropopause (−2 PVU surface) for extreme ozone events and 2 days before and 2 days after these events.

Fig. 8 shows the temporal evolution of the wave breaking through the differences of potential temperature in the tropopause between the ozone P90 days and the remaining days. The Subtropical North American wave breaking is anticyclonic (anticlockwise); that is, cold air moves equatorward and eastward (negative values in blue) to the west of warm air that moves poleward and westward (positive values in red).

Atmospheric blocking has been associated with surface extreme temperature, including heat waves, and with surface air quality (Ordóñez et al., 2017). Several studies show that traditional mid-latitude blocking results from persistent large- scale Rossby wave breaking (e.g. Polvani and Kushner, 2002; Berrisford et al., 2007; O'Kane et al., 2013; 2016). However, recently (Rodrigues and Woollings, 2017) identified subtropical South America as region where Rossby wave breaking impacts making blockings to occurs. We now examine if the Z500 differences found over central Mexico (subtropical North America) are linked to wave-breaking events. For this purpose, we used the methodology of Rodrigues and Woolings adapted from Pelly and Hoskins (2003) based on potential vorticity-potential temperature relationship. Fig. 8 shows the temporal evolution of the wave breaking through the differences of potential temperature on the tropopause between the ozone P90 days and the remaining days. The Subtropical North American wave breaking is anticyclonic (anticlockwise); that is, cold air moves equatorward and eastward (negative values in blue) to the west of warm air that moves poleward and westward (positive values in red). These composites reveal evidence of Rossby wave breaking. Therefore, this study infers that changes in large-scale atmospheric dynamics could substantially affect air quality in Mexico City.

## 4 Conclusions

In this article, the 90th percentile of Ozone concentration in Mexico City's troposphere measure by five RAMA stations was defined as the threshold for determining environmental contingencies. We study the hot-dry season form March to May of the years of stable ozone concentrations (2004 to 2018). In all cases of contingency, we found in the previous day, a stable atmosphere with almost no winds, and small fluctuations in temperature and relative humidity. Reanalysis data shows that in all the cases studied, the atmospheric blockage is due to thermal inversion by subsidence producing an accumulation of pollutants.

Ozone episodes over Mexico City are associated with elevated temperatures (exceeding 8 °C) together with air stagnation (weak winds and anticyclonic circulation in the lower troposphere). In fact, extreme ozone days over Mexico City are collocated with positive anomalies of geopotential height anomalies at 500 hPa exceeding 110 m.

In rather populated region with elevated emissions and near to mountains such Mexico City, under these stagnant synoptic conditions, the local-scale circulations of mountain flow are generated, which favor the cycling of pollutants and it precursors and therefore the photochemical production of ozone. Because of the complex terrain and atmospheric composition of Mexico City, it is possible to anticipate an environmental contingency monitoring meteorological conditions. As shown in the results, many of these air stagnation situations have their origin in atmospheric blockings, situations that can be predicted with a certain degree of certainty, when RWB occurs. We have provide a picture of the weather situations responsible for the elevated ozone concentrations over Mexico City. The findings affirm the role large-scale circulation plays on local stagnation and therefore in unhealthy air quality. However, we would like to emphasize that these results are based on composites, therefore these relationships are likely to vary for individual cases, which is a motivation for a deeper understanding of the large-scale circulation features involved in the subtropical blocking formation. In a future work we are planning to use a model like the Pasquill-Gifford (Jilani, 2009) to explain the impact of the state of thermal-dynamic atmospheric balance and the height of the mixing layer on the trigger of Ozone contingencies.

## Declarations

### Author contribution statement

Rafael Silva: Performed the experiments; Analyzed and interpreted the data; Wrote the paper.

Ana Leonor Rivera: Conceived and designed the experiments; Analyzed and interpreted the data; Wrote the paper.

Alejandro Frank: Conceived and designed the experiments; Wrote the paper.

Paulina Ordoñez Lopez, Carlos Gay: Analyzed and interpreted the data; Wrote the paper.

### Funding statement

This work was partially supported by CONACYT through Fronteras grant FC-2016-1/2277, and the Universidad Nacional Autónoma de México through DGAPA-PAPIIT IN113619 and PAPIME PE103519.

### Competing interest statement

The authors declare no conflict of interest.

### Additional information

Data associated with this study has been deposited at http://www.aire.cdmx.gob.mx.
